# Association of Monocyte Count on Admission with the Angiographic Thrombus Burden in Patients with ST-Segment Elevation Myocardial Infarction Undergoing Primary Percutaneous Coronary Intervention

**DOI:** 10.5935/abc.20180034

**Published:** 2018-04

**Authors:** Zuoyan Wang, Na Liu, Lihui Ren, Licheng Lei, Huiming Ye, Jianjun Peng

**Affiliations:** Beijing Shijitan Hospital - Capital Medical University

**Keywords:** Myocardial Infarction, Percutaneous Coronary Intervention/methods, Coronary Thrombosis/diagnostic imaging, Monocytes

## Abstract

**Background:**

The intracoronary high-thrombus burden during the primary percutaneous coronary intervention in patients with ST-elevation myocardial infarction (STEMI) can lead to poor outcomes. Monocytes have been described to play an important role in thrombotic disorders.

**Objectives:**

This study aimed to investigate the relationship between admission monocyte count and angiographic intracoronary thrombus burden in patients receiving primary percutaneous coronary intervention (PPCI).

**Methods:**

A total of 273 patients with acute STEMI who underwent PPCI were enrolled. The patients were divided into two groups according to the thrombolysis in myocardial infarction (TIMI) thrombus grade: low-thrombus burden group with a grade of 0-2 and high-thrombus burden group with a grade of 3-4. The monocyte count and other laboratory parameters were measured on admission before PPCI. P-value < 0.05 was considered significant.

**Results:**

There were 95 patients (34.8%) in the high-thrombus burden group, and 178 patients (65.2%) in the low-thrombus burden group. Patients with high-thrombus burden had significantly higher admission monocyte count (0.61 ± 0.29×10^9^/L vs. 0.53 ± 0.24×10^9^/L, p = 0.021). In multivariate analysis, monocyte count was the independent predictor of angiographic high-thrombus burden (odds ratio 3.107, 95% confidence interval [CI] 1.199-7.052, p = 0.020). For the prediction of angiographic high-thrombus burden, admission monocyte count at a cut-off value of 0.48×10^9^/L yielded 0.59 ROC-AUC (71.9% sensitivity, 46.9% specificity).

**Conclusions:**

Monocyte count on admission was an independent clinical predictor of high-thrombus burden in patients with STEMI undergoing PPCI. Our findings suggest that admission monocyte count may be available for early risk stratification of high-thrombus burden in acute STEMI patients and might allow the optimization of antithrombotic therapy to improve the outcomes of PPCI.

## Introduction

Complete thrombotic occlusion of a major epicardial coronary artery is the common pathophysiological mechanism of acute ST-segment elevation myocardial infarction (STEMI). Primary percutaneous coronary intervention (PPCI) of the infarct-related artery in patients with STEMI is associated with prompt restoration of normal blood flow and improved clinical outcomes. However, clinical studies have shown that a high burden of peri-procedural intracoronary thrombus is an essential contributor of low thrombolysis in myocardial infarction (TIMI) flow grade and impaired myocardial perfusion. Identification of relationships between blood cell-related biomarkers and blood flow state during PPCI procedure is one of the current research focuses. Studies have revealed that monocytes may be involved in the pathogenesis of coronary artery disease^1^ and elevated monocyte count is a risk factor for myocardial infarction.^2^ Previous studies have shown that monocytes play an important role in thrombotic disorders, not only via the secretion of pro-coagulant factors, such as tissue factor, but also by promoting inflammation processes. In our previous reports, monocyte counts on admission independently predict no-reflow following primary PPCI.^3^ In the current study, we aimed to investigate further the relationship between on admission monocyte count and angiographic intracoronary thrombus burden in patients receiving PPCI.

## Methods

### Study population

We enrolled 273 consecutive patients with STEMI undergoing PPCI within 12h from symptom onset between September 2013 and May 2016 at the Cardiology Department of Beijing Shijitan Hospital. STEMI was defined as: typical chest pain > 30 minutes with ST elevation of > 1 mm in at least two consecutive leads on the electrocardiogram, or new onset left bundle branch block and more than two-fold increase in serum cardiac markers. Exclusion criteria included cardiogenic shock on admission, active infections, systemic inflammatory disease history, known malignancy, liver disease, as well as renal failure. The study protocol was approved by the Beijing Shijitan Hospital Ethics Committee, Capital Medical University, and written informed consent was obtained from patients.

### Coronary angiography and PCI procedure

Pharmacological treatment of all enrolled patients before PPCI included aspirin (300 mg loading dose), clopidogrel (600 mg loading dose) and an intravenous bolus of unfractionated heparin at a dose of 70 U/kg of body weight. PPCI was performed using the standard radial or femoral approach with a 6-or 7-French guiding catheter. The stent was deployed in all patients. The use of balloon pre-dilatation or post-dilatation, the type of stents (bare metal or drug-eluting), and the use of thrombus aspiration was left to the operator's decision. The glycoprotein IIb/IIIa receptor inhibitor tirofiban was given by judgment of the operator and initiated during PCI procedure with 10 µg/kg intracoronary bolus followed by 0.15 µg/kg/min intravenous infusion. Technically successful stent implantation was defined as the residual stenosis < 10% in the culprit lesion after the procedure as visually assessed by angiography, without occlusion of a significant side branch, flow-limiting dissection, distal embolization, or angiographic thrombus.

To evaluate the intracoronary thrombus burden we performed TIMI thrombus scale^4,5^ in all patients after ante-grade flow achievement through guide wire crossing or small balloon dilatation (final TIMI thrombus grade). In TIMI thrombus grade 0, no cine-angiographic characteristics of thrombus are present; in TIMI thrombus grade 1, possible thrombus is present with such angiographic characteristics as decreased contrast density, haziness, irregular lesion contour, or a smooth convex "meniscus"at the site of total occlusion suggestive, but not diagnostic of thrombus; in TIMI thrombus grade 2, there is definite thrombus, with the largest dimensions ≤ 1/2 the vessel diameter; in TIMI thrombus grade 3, there is definite thrombus with the largest linear dimension > 1/2 but < 2 vessel diameters; in TIMI thrombus grade 4, there is definite thrombus, with the largest dimension ≥ 2 vessel diameters; and in TIMI thrombus grade 5, there is total occlusion. Patients were divided into two groups according to the final TIMI thrombus grade: low-thrombus burden group with a grade of 0-2, and high-thrombus burden group with a grade of 3-4.

### Laboratory analysis and echocardiography

In all patients, blood samples for measurements were performed according to our previous work.^3^ White blood cell (WBC) count, monocyte count, and other biochemical parameters were drawn into standard ethylenediaminetetraacetic acid (EDTA) containing tubes on admission in the emergency room before the administration of aspirin and clopidogrel. Common blood counting (CBC) parameters were measured by an automated blood cell counter (XS-1000i; Sysmex Co.). Creatinine and cardiac enzymes were also measured in all patients determined by the standard methods. Echocardiography investigation was routinely performed on admission before PPCI, using GE ViVidE7 ultrasound machine (GE Healthcare, America) with a 3.5-MHz transducer. Left ventricular ejection fraction (LVEF) was measured by Simpson's method in the 2-dimensional echocardiographic apical 4-chamber view.

### Statistical analyses

Statistical analysis was performed by using the SPSS 22.0 Statistical Package Program for Windows (SPSS Inc., Chicago, IL, USA). Continuous variables are presented as a mean ± standard deviation or as medians and interquartile ranges. The differences between groups of continuous variables with a normal distribution (age, LVEF, creatinine, stent parameters and hematological parameters) were tested by independent samples t-test, while skewed distribution variable (peak cardiac troponin I (cTnI)) were compared by the Mann-Whitney U test. Categorical variables were summarized as percentages and compared with the chi-square test. A univariate analysis was first performed to test for the association of the high-thrombus burden and several potentially impacting variables (age, sex, history of diabetes mellitus, prior myocardial infarction (MI), LVEF, creatine level, time from symptom onset to PPCI, monocyte count, neutrophil count, lymphocyte count and hemoglobin level). Multivariate logistic regression analysis was then used to identify independent predictors of high thrombus burden using variables (prior MI, time from symptom onset to PPCI and monocyte count) that reached a trend-level effect (p < 0.1) in the univariate analyses. The receiver operating characteristics (ROC) curve was used to determine the cut-off value of monocyte count to predict the high-thrombus burden. A two-sided p-value of < 0.05 was considered significant.

## Results

A total of 273 patients (mean age 62.2 ± 13.6 years; 81.0% male) who underwent PPCI were enrolled in our analysis. Stent implantation was technically successful in all patients. The comparison of baseline clinical and laboratory characteristics between thrombus burden groups are presented in Table 1. There were no significant differences between the low thrombus group and the high thrombus group in the age, sex distribution, hypertension, diabetes mellitus, hyperlipidemia, current smoking, prior MI left ventricular ejection fraction and serum creatinine level. Compared with patients with low thrombus burden, patients with high-thrombus burden had higher peak cTnI.

**Table 1:** Baseline clinical and laboratory characteristics of the study population divided according to thrombus burden

	Low thrombus burden (n = 178)	High thrombus burden (n = 95)	p value
Age (years)	62.3 ± 13.2	62.0 ± 14.7	0.866*
Male sex, n (%)	143 (80.3)	77 (81.1)	0.482†
Diabetes mellitus, n (%)	56 (31.5)	31 (32.6)	0.716†
Hypertension, n (%)	106 (59.6)	57 (60.0)	0.503†
Hyperlipidemia, n (%)	109 (61.2)	65 (68.4)	0.395†
Active smokers, n (%)	75 (42.1)	47 (49.5)	0.200†
Prior MI, n (%)	6 (3.4)	3 (3.2)	0.145†
LVEF (%)	53.4 ± 9.7	52.9 ± 8.5	0.699*
Creatinine, µmol/L	76.9 ± 24.5	81.4 ± 25.6	0.167*
Peak cTnI (ng/mL)	29.8 (1.2-86.6)	56.7 (16.4-100.6)	0.037‡

* Independent samples t-test;

† Chi-square test;

‡ Mann-Whitney U test; MI: myocardial infarction; LVEF: left ventricular ejection fraction; cTnI: cardiac troponin I.

Comparison of the baseline angiographic and procedural characteristics of the groups based on thrombus burden is shown in Table 2. Thrombus aspiration device and intracoronary tirofiban administration were used more frequently in the high thrombus burden group than low-thrombus burden group (62.1 vs. 10.1%, p = 0.000, 83.2 vs. 52.2%, p = 0.000, respectively). There were no significant differences of the time from pain to intervention, infarct-related coronary artery and other procedural characteristics between two groups.

**Table 2:** Baseline angiographic and procedural characteristics according to thrombus burden

Variable	Low thrombus burden (n = 178)	High thrombus burden (n = 95)	p value
Time from symptom onset to PPCI			0.773^†^
< 3 h (%)	48 (26.9)	27 (28.4)	
3-6 h (%)	66 (37.1)	38 (40.0)	
6-12 h (%)	64 (36.0)	30 (31.6)	
Anterior infarct location, n (%)	95 (53.4)	48 (50.5)	0.918^†^
**Infarct-related coronary artery, n (%)**			**0.788^†^**
Left main	0 (0.0)	0 (0.0)	
Left anterior descending	95 (53.4)	49 (51.6)	
Left circumflex	22 (12.4)	12 (12.6)	
Right coronary artery	61 (34.2)	34 (35.8)	
Number of used stent, n	1.6 ± 0.7	1.4 ± 0.7	0.106*
Total stent length, (mm)	36.7 ± 19.1	36.6 ± 17.6	0.164*
Stent diameter, (mm)	3.1 ± 0.4	3.2 ± 0.5	0.164*
Use of thrombus aspiration, n (%)	18(10.1)	59 (62.1)	0.000^†^
Tirofiban use, n (%)	93 (52.2)	79 (83.2)	0.000^†^

* Independent samples t-test;

† Chi-square test.

The comparison of admission hematological parameters is presented in Table 3. WBC counts, neutrophil counts, platelet count, hemoglobin, hematocrit, mean platelet volume, and lymphocyte count were similar in both groups. The patients in the high-thrombus burden group had significantly higher monocyte count when compared with the patients of low-thrombus burden group (0.61 ± 0.29×109/L vs. 0.53 ± 0.24×109/L, p = 0.021.

**Table 3:** Hematological parameters of the study population

Variable	Low thrombus burden (n = 178)	High thrombus burden (n = 95)	p value
White blood cell count ×10^9^/L	9.6 ± 3.0	9.9 ± 3.2	0.326*
Neutrophil count×10^9^/L	6.8 ± 2.8	6.9 ± 3.3	0.774*
Hemoglobin g/dL	14.4 ± 1.9	14.4 ± 2.3	0.707*
Platelet count×10^9^/L	214.3 ± 60.5	218.8 ± 53.8	0.551*
Hematocrit %	42.3 ± 4.7	42.2 ± 4.9	0.835*
Mean platelet volume fl	10.3 ± 0.8	10.2 ± 0.9	0.668*
Lymphocyte count×10^9^/L	2.23 ± 1.94	2.32 ± 1.35	0.827*
Monocyte count ×10^9^/L	0.53 ± 0.24	0.61 ± 0.29	0.021*

* Independent samples t-test.

Univariate and multivariate logistic regression analysis of the association between the angiographic high thrombus burden and multiple parameters are presented in Table 4.

**Table 4:** Independent predictors of high-thrombus burden in patients with ST-elevation myocardial infarction in logistic regression analyses

Variable	Univariate		Multivariate	
	Odds ratio (95%CI)	p value	Odds ratio (95%CI)	p value
Age	0.998(0.980-1.016)	0.829		
Sex	1.033(0.549-1.943)	0.921		
Diabetes mellitus	0.932(0.547-1.588)	0.797		
Prior MI	1.869 (0.781-9.178)	0.092	1.745 (0.752-8.644)	0.495
LVEF	0.995 (0.968-1.022)	0.702		
Time from symptom onset to PPCI	1.021 (1.008-1.208)	0.094	1.002 (0.979-1.195)	0.553
Creatinine	1.007 (0.997-1.017)	0.194		
Neutrophil count	1.016 (0.935-1.104)	0.704		
Hemoglobin	0.998 (0.986-1.010)	0.780		
Lymphocyte count	1.019 (0.886-1.173)	0.790		
Monocyte count	2.429 (1.022-5.776)	0.045	3.107 (1.199-7.052)	0.020

MI: myocardial infarction; LVEF: left ventricular ejection fraction; PPCI: primary percutaneous coronary intervention.

In multivariate analyses, on admission monocyte count was an independent predictor of angiographic high thrombus burden (odds ratio 3.107, 95% confidence interval [CI] 1.199-7.052, p = 0.020). The most discriminative cut-off values of monocyte count were 0.48×10^9^/L, with a sensitivity of 71.9% and a specificity of 46.9% (AUC: 0.59; 95% CI: 0.515-0.654; p = 0.035).

## Discussion

Acute STEMI is characterized by complete thrombotic occlusion of a coronary artery. The goal of PPCI in STEMI is the rapid restoration of coronary blood flow, maximum salvage of myocardium and improving patients' outcomes after STEMI. Studies have shown that intracoronary thrombi can lead to micro- and macro-vasculature embolization and is associated with poor outcomes in patients who underwent PPCI of culprit lesion.^6-8^ However, management of thrombotic burden is still challenging during PPCI for STEMI. Early risk stratification to detect patients at high risk of high thrombus burden is very important for the individualized prevention and treatment of this condition. In the current study, elevated admission monocyte counts were found as an independent predictor of high thrombus burden of infarct-related artery (IRA) during PPCI in patients with STEMI.

Inflammation and oxidative stress were found to play an important role in the pathogenesis of plaque rupture and subsequent thrombus formation.^9,10^ Monocyte comprises 10% of human blood leukocytes and is one of the major players of systemic inflammatory response. They are associated with the inflammatory response at the vulnerable plaque in patients with STEMI.^11^ Tissue factor (TF) is an essential component of the extrinsic coagulation cascade and is critical in arterial thrombosis. Recent data have suggested that monocytes appear to be the major source of blood TF.^12^ Palmerini et al.,^13^ conducted a histological evaluation of thrombi aspirated from coronary arteries of patients with STEMI and found that monocytes consistently stained strongly for tissue factor, while neutrophils had a weakly and irregular tissue factor staining. Another explanation for the relationship between monocytes and high thrombus burden may be the increased formation of monocyte-platelet aggregates (MPA). MPA is a useful marker of platelet activation in ACS patients^14^ and was found to be a significant predictor of no-reflow in patients with STEMI undergoing primary PCI.^15^

Involvement of monocytes in the prothrombotic state is not restricted to the above-mentioned mechanisms. Aleman et al.,^16^ showed that microparticles (MPs) from monocytes are associated with prothrombinase activity and faster fibrin formation. Furthermore, monocytes may induce thrombus generation by promoting inflammation processes. Mach et al.,^17^ found that stimulation of human monocytes induced the expression of interstitial collagenase and stromelysin, which were associated with plaque destabilization and thrombotic events. Post-mortem investigation and histological studies of in vivo-obtained thrombus specimens of STEMI patients revealed that approximately 50% of the aspirated thrombi were days to even weeks old,^18^ which suggests that prothrombotic factors, such as elevated levels of circulating monocyte, may starts days or even weeks before symptom onset during STEMI.

In the current study, admission monocyte count was evaluated regarding its potency to differ between high thrombus burden and low thrombus burden in STEMI patients underwent PPCI. However, admission monocyte count at cut-off value of 0.48×10^9^/L presented a low diagnostic performance with 71.9% sensitivity and 46.9% specificity. A combination of parameters, including monocyte count, may be needed to improve diagnostic abilities.

The main limitations of this study were the retrospective design and relatively small number of patients. Also, the prior antithrombotic therapeutic profiles of the study population, which might affect intracoronary thrombotic status during PPCI, were not routinely available in the present study and not included in the risk factor analysis. Further large prospective cohort study with assessment of detailed information of prior antithrombotic therapy might be more illuminating.

## Conclusions

In conclusion, we found that monocyte count on admission, which is cheaply and easily measurable laboratory data, is a predictor of high intracoronary thrombus burden in patients with STEMI undergoing primary PCI.
